# Supplementary material for: DMRforPairs: identifying Differentially Methylated Regions between unique samples using array based methylation profiles
Source: BMC Bioinformatics. 2014 May 15;15:141. doi: 10.1186/1471-2105-15-141 (PMC4046028; doi:10.1186/1471-2105-15-141)
Supplement: Additional file 2 — DMRforPairs output for the comparison of A431-MCF7 and NA17018-NA17105. Please start from the HTML files in each folder. Available via the BMC Bioinformatics website. [file 1471-2105-15-141-S2.zip › 1394847754114233_MOESM2_ESM/A431_MCF7/figures/26102.pdf]

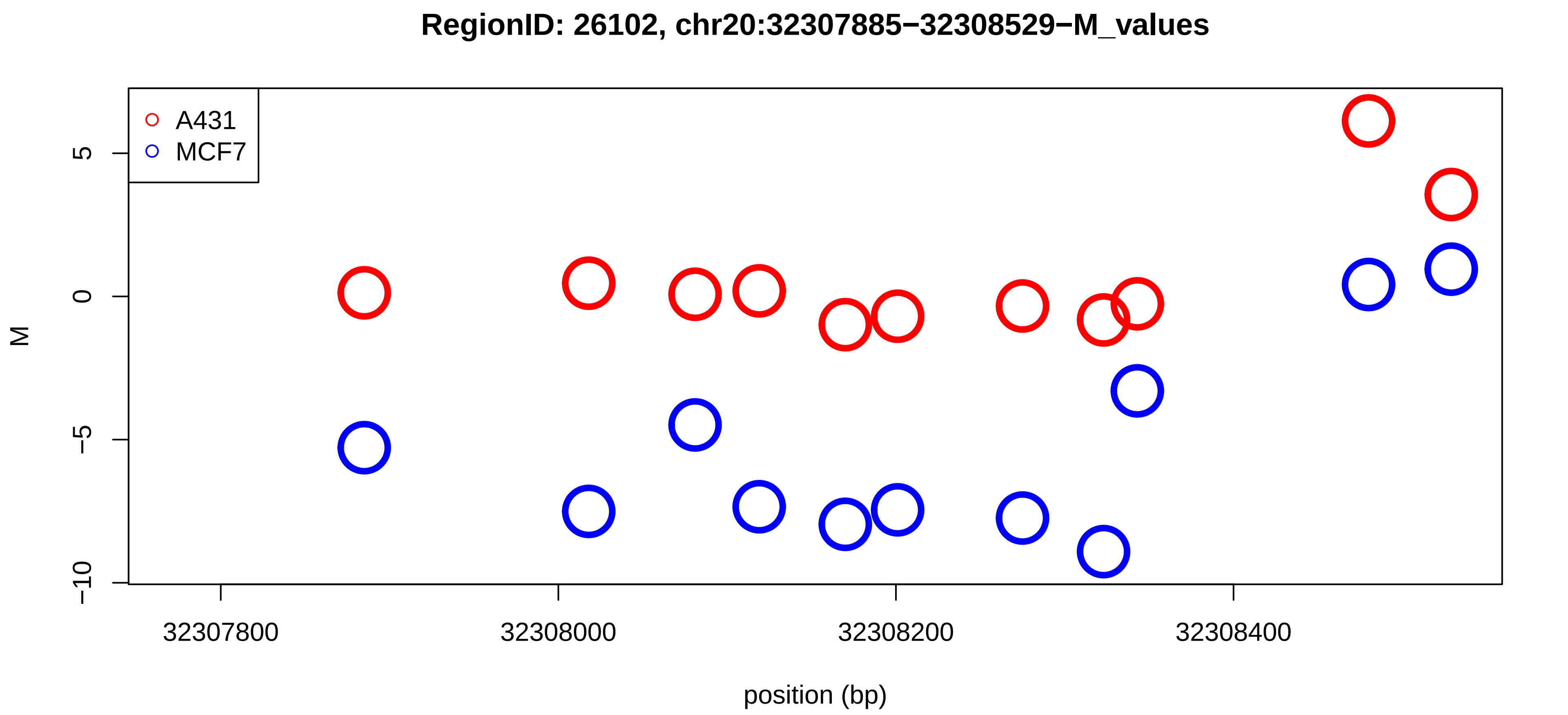
RegionID: 26102, chr20:32307885−32308529−M_values
A431
MCF7
M
position (bp)
32307800
32308000
32308200
32308400
5
0
−5
−10

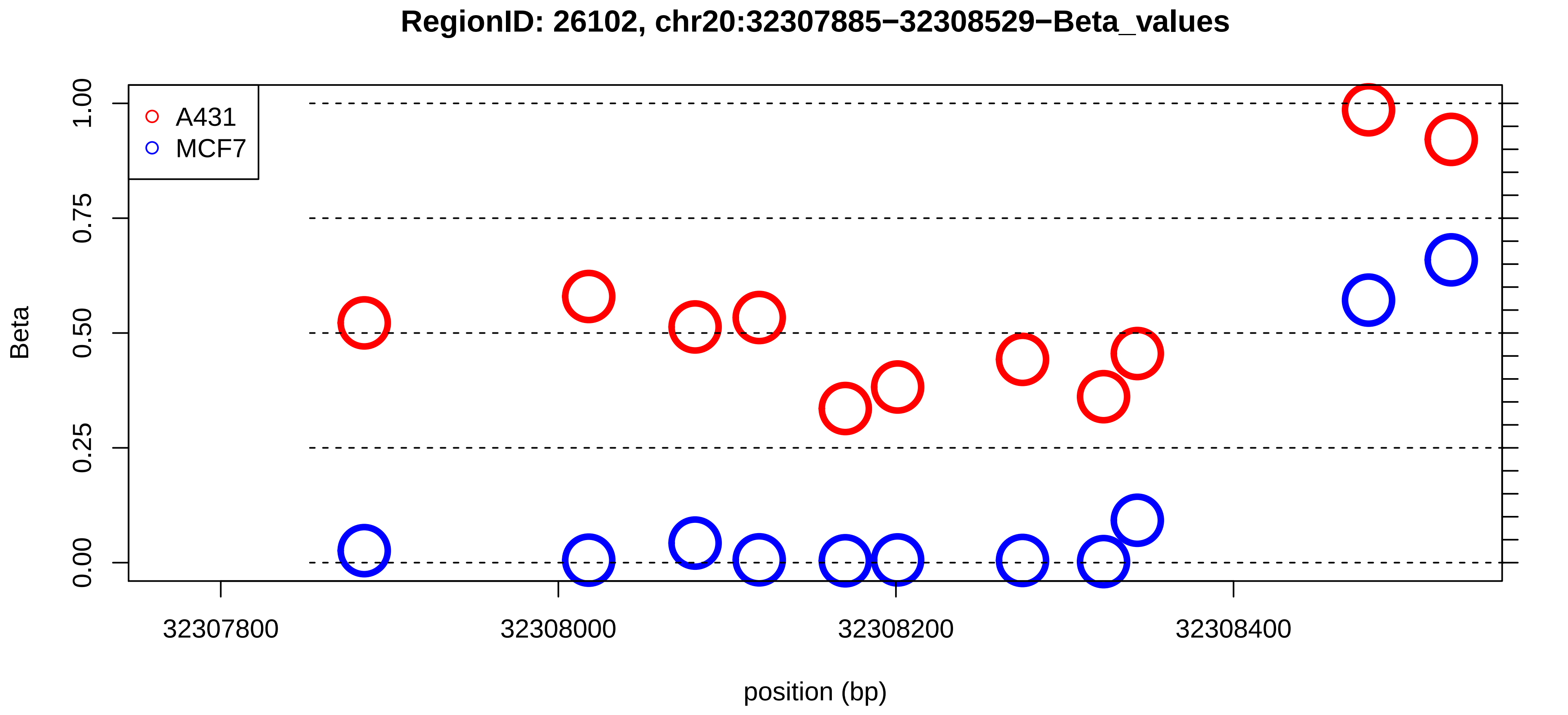
RegionID: 26102, chr20:32307885−32308529−Beta_values
A431
MCF7
Beta
position (bp)
32307800
32308000
32308200
32308400
1.00
0.75
0.50
0.25
0.00

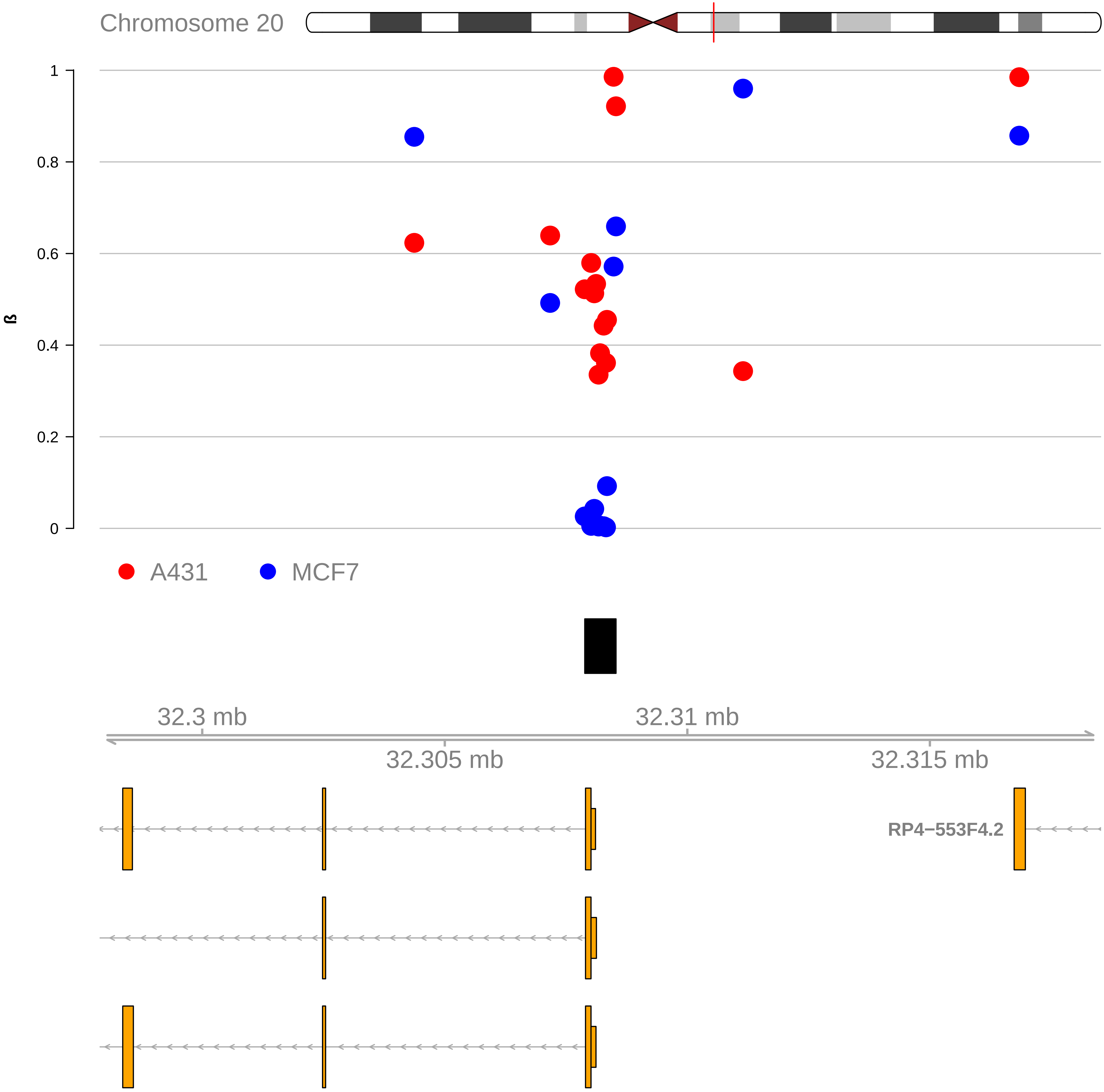
Chromosome 20
1
0.8
0.6
0.4
0.2
0
ß
A431
MCF7
32.3 mb
32.31 mb
32.305 mb
32.315 mb
RP4−553F4.2
